# Freestanding bilayer plasmonic waveguide coupling mechanism for ultranarrow electromagnetic-induced transparency band generation

**DOI:** 10.1038/s41598-021-81118-6

**Published:** 2021-01-14

**Authors:** Li Yu, Yuzhang Liang, Shuwen Chu, Huixuan Gao, Qiao Wang, Wei Peng

**Affiliations:** 1 School of Physics, Dalian University of Technology, Dalian, 116024 China; 2 School of Optoelectronic Engineering and Instrumentation Science, Dalian University of Technology, Dalian, 116024 China

**Keywords:** Nanoscience and technology, Nanoscale devices

## Abstract

Strong electromagnetic coupling among plasmonic nanostructures paves a new route toward efficient manipulation of photons. Particularly, plasmon-waveguide systems exhibit remarkable optical properties by simply tailoring the interaction among elementary elements. In this paper, we propose and demonstrate a freestanding bilayer plasmonic-waveguide structure exhibiting an extremely narrow transmission peak with efficiency up to 92%, the linewidth of only 0.14 nm and an excellent out of band rejection. The unexpected optical behavior considering metal loss is consistent with that of electromagnetic induced transparency, arising from the destructive interference of super-radiative nanowire dipolar mode and transversal magnetic waveguide mode. Furthermore, for slow light application, the designed plasmonic-waveguide structure has a high group index of approximately 1.2 × 10^5^ at the maximum of the transmission band. In sensing application, its lowest sensing figure of merit is achieved up to 8500 due to the ultra-narrow linewidth of the transmission band. This work provides a valuable photonics design for developing high performance nano-photonic devices.

Plasmonic nanostructures based on resonant coupling and hybridization phenomenon have exhibited a variety of intriguing optical attributes and functionalities^1–3^. The exploration of exotic optical properties in well-designed plasmonic coupling systems has been the subject of extensive research efforts for inspiring the development of nano-optical devices, such as nanolaser^4, 5^, biosensors^6–8^, optical imaging filter^9–11^, photovoltaics^12^ and the like. However, the performance of plasmonic systems is commonly restricted by broad spectrum feature mainly stemming from the strong radiative damping and intrinsic damping of metal loss. One effective approach around the issue is to employ the coupling or hybridization of plasmonic nanostructures to elaborately engineer the resonant mode of hybridized systems^12, 13^. Currently, all-optical Fano resonance and electromagnetic-induced transparency (EIT) as two typical coupled plasmonic modes, have been investigated extensively for many practical applications^1, 14–16^. The most representative example of plasmonic hybridized systems is the periodically arranged metallic nanowire pairs^17, 18^. The strong interplay between asymmetric nanowire pairs results in the formation of two distinct plasmonic modes: broad band symmetric mode and narrow band antisymmetric mode, the coupling of which results in a Fano resonance and greatly suppresses the radiative damping of plasmonic nanostructures to form narrow band sub-radiative mode. Another prominent example is the combination of periodic gold nanowires with a slab waveguide structure for generating the EIT transmission peak^19–21^, which is attributed to the coherent coupling of nanowire plasmonic mode and optical waveguide mode. However, there are few reports to combine these above two common hybridized structures together to construct a bilayer plasmonic waveguide system^11, 22, 23^. In contrast to isolated hybridized structures, the interaction between waveguide mode and two hybridized modes between nanowire pairs can lead to a very pronounced modification of their optical response.

In the paper, we provide an extremely narrow EIT transmission band by coupling optical waveguide mode to the broad low-transmission spectrum of plasmonic nanowire pairs in a free-standing bilayer plasmonic waveguide structure. The longitudinal symmetric waveguide structure benefited from the absence of substrate eliminates cut-off thickness for guided mode, which significantly decreases the line width of transmission peak with thin waveguide thickness. We systematically investigate the modification of the lateral displacement along two interacting nanowires on the optical response of the proposed hybridized structure. It is shown that the spectrally optical response is prominently modified by additional magnetic resonant modes from strong near-field interactions between nanowires. The proposed plasmonic waveguide structure is also verified in principle for potential applications of slow light and sensing.

## Principle design and mechanism operation

**Figure 1:**
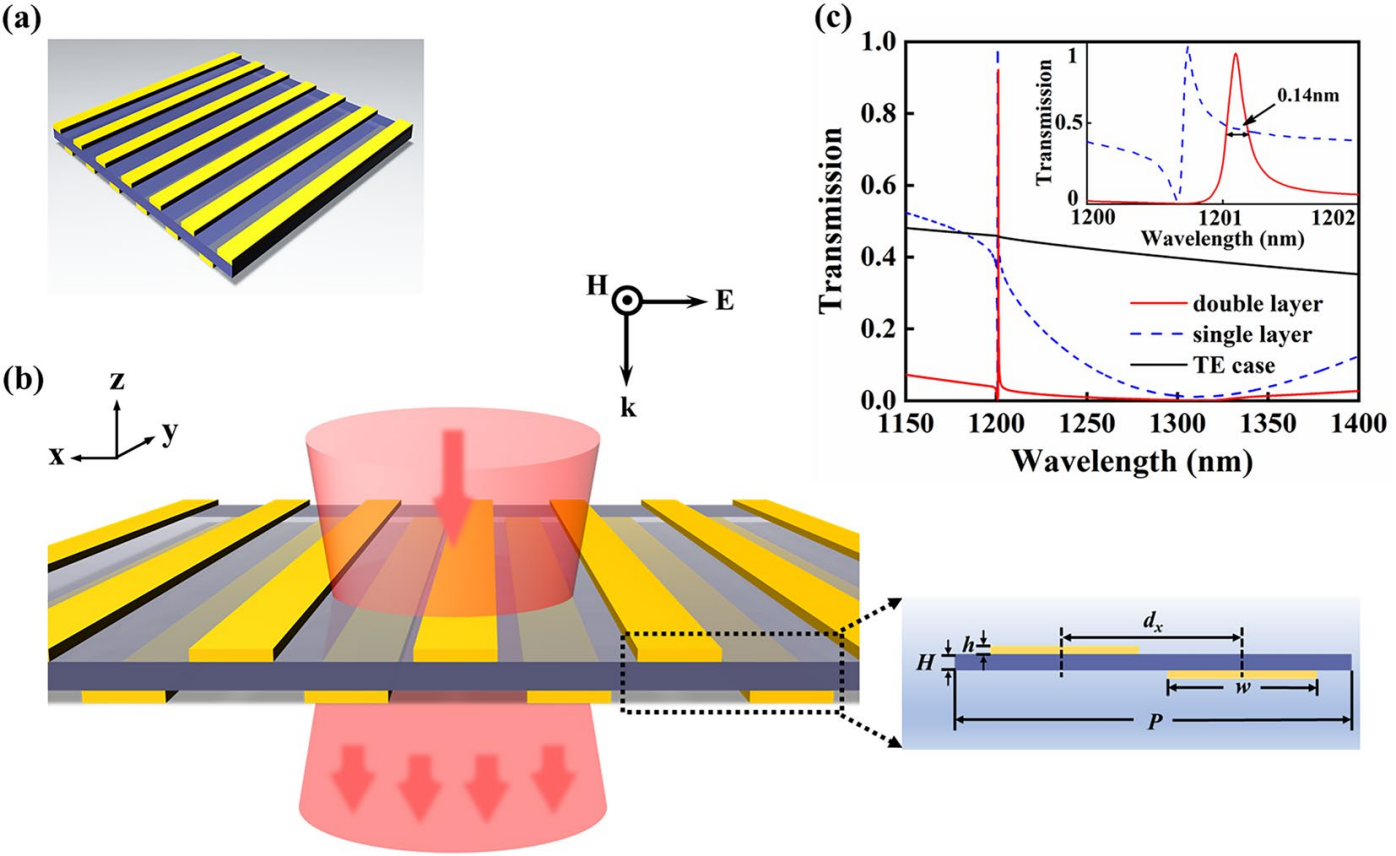
Freestanding bilayer plasmonic waveguide structure and its representative optical characteristics. (**a**) Three-dimensional schematics of the proposed plasmonic nanostructure consisting of two identical gold nanowire gratings separated by a finite dielectric waveguide and a lateral shift between them. (**b**) Front view of the proposed nanostructure. Inset shows the cross section of a structure unit cell with geometric dimensions specified. (**c**) The transmission (red solid line) spectrum for the plasmonic waveguide structure with waveguide thickness *H* = 20 nm and the lateral shifts *d*_*x*_ = 550 nm. As a contrast, the transmission spectrum (blue dashed line) of single nanowire grating and waveguide structure is also shown for TM polarization and normal light incidence. In addition, transmission spectrum of double layer structure for TE polarization is also illustrated by black solid line. Inset shows a magnified view of transmission band for above two structures, in which the bandwidth and spectral features of transmission peak are legible.

Figure 1 illustrates this freestanding bilayer plasmonic waveguide structure and its representative optical characteristics. As shown in Fig. 1a, b, the studied plasmonic-waveguide structure consists of two identical nanowire gratings separated by a finite silica slab waveguide without any substrate below the structure. The inset of Fig. 1b shows the cross section of a structure unit cell. Compared to single layer nanowire gratings, each unit cell contains two nanowires regarded as a plasmonic superlattice, the coupling of which and the length of each nanowire determines the frequency of plasmon mode, related directly to the line shape of the optical spectra. The broken of structural symmetry is introduced by a lateral shift *d*_*x*_ in a unit cell. The nanowire has a cross section of 450 × 10 nm^2^ and is made of gold. Because the plasmonic resonance of each nanowire is proportional to the nanowire length, the nanowire length is specified by *w* = 450 nm in the considered infrared spectrum region. The nanowire period of *P* = 1200 nm is kept constant. The period is chosen to provide the necessary momentum to excite the waveguide mode by coherent plasmonic scattering of gold nanowires in the infrared region. Therefore, for the fixed thickness waveguide of *H* = 20 nm, the frequency of waveguide mode is determined by period *P*. Remarkably, there are no frequency cut-offs for guided mode at any waveguide layer thickness due to the absence of waveguide substrate or in a symmetric waveguide structure. Numerical calculation is performed by utilizing a finite different-time domain (FDTD) method^24^, which is generally well-suited for calculating the optical properties of complex metallic nanostructures. For all calculations, the propagation of incident light is in the normal (− *z*) direction with magnetic field parallel to the nanowires. The periodic boundary is used in *x* direction and the perfect matched layer condition is applied for *z* direction. The dielectric permittivity of silica is 2.13 and that of gold is described by Drude-Lorentz model^25^. All calculations with extremely well convergence conditions are performed with a fine mesh of 0.5 × 0.5 nm^2^. In our calculations, only the case of transverse magnetic (TM) polarized light (magnetic field parallel to nanowire) is considered. As depicted in the black solid line of Fig. 1c, there is no distinct transmission peak under transverse electric (TE) polarized light, which can be attributed to the following aspects: firstly, the plasmonic mode of metal gratings cannot be excited under TE polarized light; secondly, under the existing structure parameters, the coupling between TE polarized light and the proposed bilayer plasmonic waveguide structure is too weak to excite narrowband TE guided mode in the waveguide slab. That is, narrowband transmission peak might be excited with appropriate structure parameters under TE polarized light. The proposed plasmonic waveguide device is easy to fabricate by the combination of electron beam evaporation and focus ion beam milling techniques on a SiO_2_ membrane window^23^.

## Results and discussion

As illustrated in Fig. 1c, the coupling of plasmonic modes of nanowire pairs with a lateral shift *d*_*x*_ = 550 nm and the waveguide mode results in a distinct transmission spectrum (red solid line). An extremely narrow and high transmission peak with only 0.14 nm linewidth is observed with the broad-spectrum region. Obviously, this unique transmission property originates from the spectra overlap of the narrow transmission band of waveguide mode and the broad plasmonic scattering band of nanowire pairs mode. The narrow transmission band arises from the excitation of the waveguide mode (i.e. optically dark mode) through the scattering of localized plasmonic mode in the metal nanowire pairs. And the broad plasmonic band is directly excited by the localized plasmonic mode in the metal nanowire pairs. Hence, the spectral position and line width of the transmission band are determined by the coupling strength between these above two modes. To obtain the narrow transmission band, the thickness of waveguide layer is chosen to be thin. Meanwhile, the transmission peak shifts to a shorter wavelength with the reduction of the thickness of the waveguide layer. In our analysis, the position of the transmission peak is approximately at the period of nanowire grating. Noteworthy, here the coupling with waveguide mode results in the transmission peak rather than the extinction peak of Rayleigh anomaly^26^.

For a direct comparison, the transmission spectrum for the plasmonic-waveguide system with single layer nanowire grating is also shown by the blue dashed line of Fig. 1c. The inset of Fig. 1c clearly demonstrates the difference between two EIT transmission bands between single-layer and double-layer nanowire structures. Apparently, the EIT transmission band for the double-layer structure has a weakly asymmetric line shape with the sideband transmission below 5% in the wavelength range from 1150 to 1400 nm, which is highly desirable for the applications of slow light and high-sensitivity sensing. A most striking distinction is that the double-layer structure possesses a broad radiative plasmon mode in comparison with the case of the single-layer structure due to the coupling of two nanowires in an individual unit cell, which can be understood in terms of plasmon hybridization.

**Figure 2:**
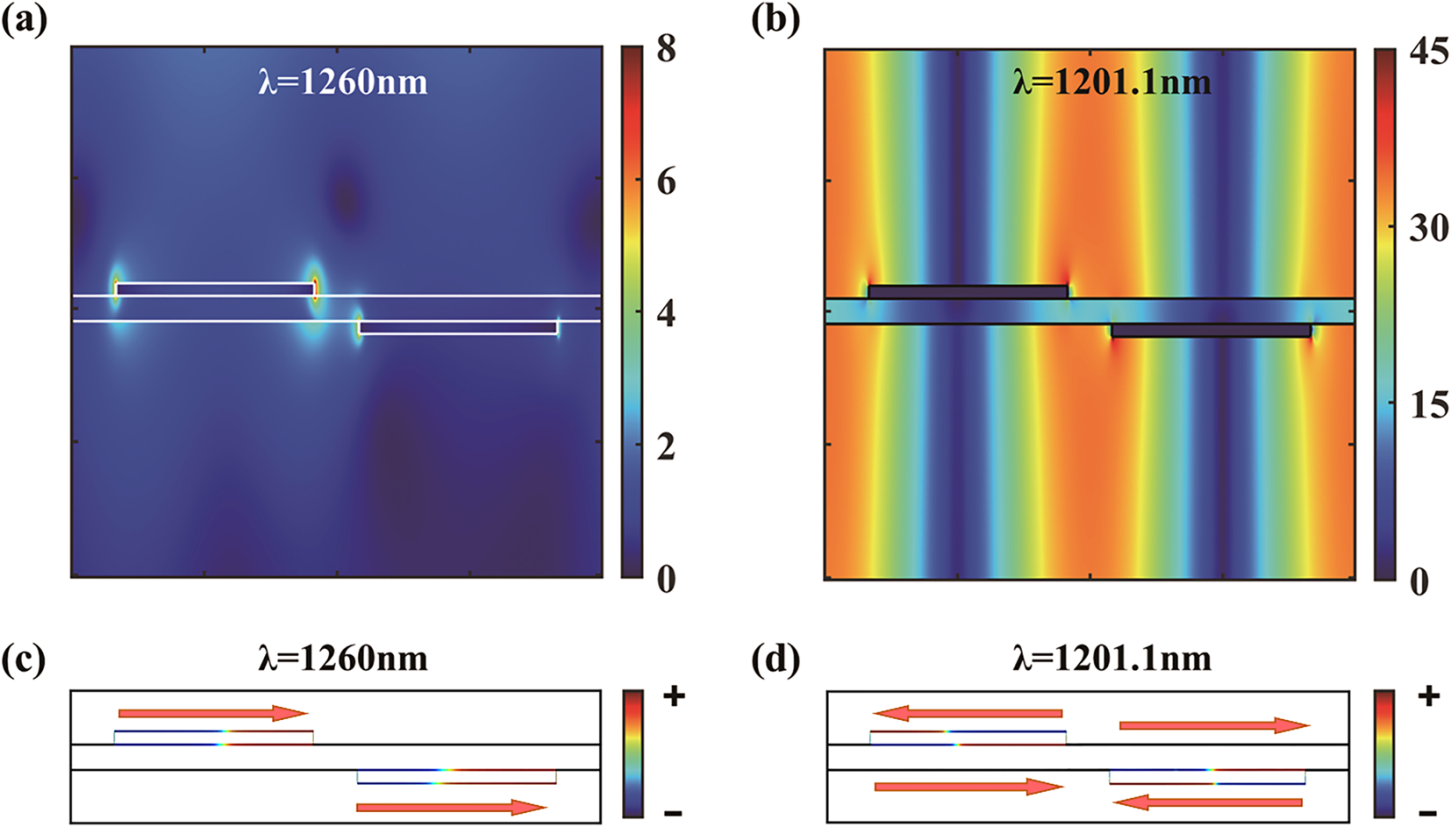
Calculated spatial distribution of magnitude of the electric field and corresponding charge distribution for two different wavelengths. (**a**) The electric field and (**c**) the charge for a somewhat longer wavelength of λ = 1260 nm where the reflection has a maximum corresponding to super-radiant mode. (**b**) The electric field and (**d**) the charge at the transmission peak of λ = 1201.1 nm corresponding to sub-radiative mode. The solid lines in panels (**a**) and (**b**) indicate the interfaces of structure.

**Figure 3:**
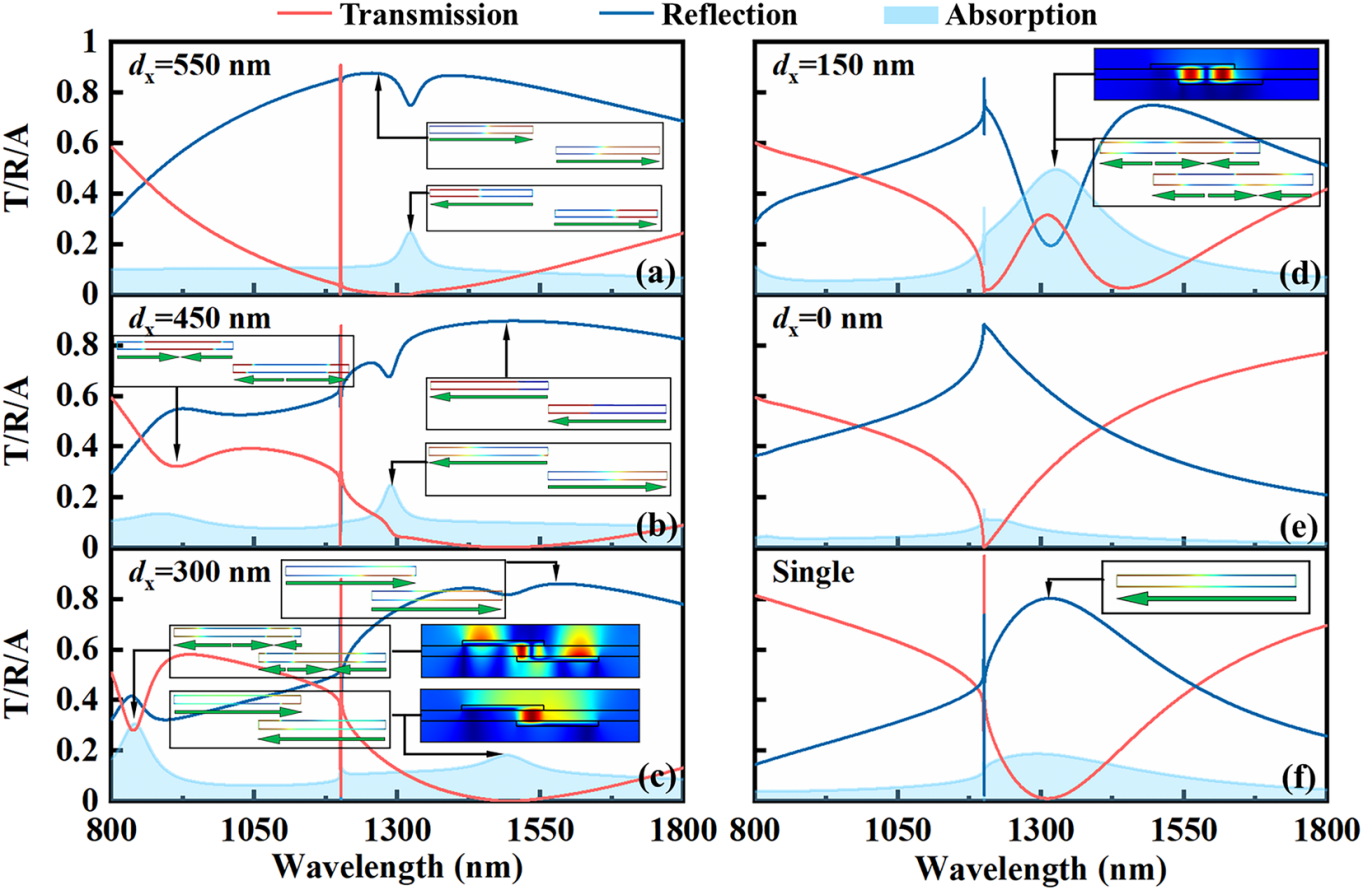
The transmission (red solid lines), reflection (blue solid lines) and absorption (cyan shaded area) spectra of the plasmonic waveguide structure for TM polarization and normal light incidence with the lateral shift *d*_*x*_ = 550 nm (**a**), 450 nm (**b**), 300 nm (**c**), 150 nm (**d**), and 0 nm (**e**). The charge and magnetic field distributions at some specified locations are shown in the inset for clearly determining the origins of resonate modes. The transmission, reflection and absorption spectra of the structure consisting of single layer nanowire grating and waveguide are given as a direct comparison in panel (**f**). Insets in panel (**f**) corresponds to the charge distribution of reflection peak.

We present the calculated spatial distribution of magnitude of the electric field and corresponding charge distribution for two different wavelengths in Fig. 2. Figure 2a illustrates the local electric field distribution of super-radiant plasmon mode in a unit cell at the wavelength of 1260 nm (maximum of reflection), where strong near field is mainly localized around the two nanowires. The super-radiant mode is characterized in Fig. 2c by the in-phase oscillation of the free electrons along these two interacting nanowires, namely, in-phase first-order dipolar distribution. This mode increases the appearing restoring force and radiative damping, and therefore increases the energy and spectral bandwidth, leading to the blue-shifted broad super-radiant broad spectrum compared with the dipolar mode of the individual nanowire at 1315 nm (in Fig. 3f). Therefore, the weakly asymmetric line shape of the EIT transmission band in the double-layer structure is due to the broad and low transmission of symmetric super-radiant plasmon mode. In this case, the corresponding sub-radiant antisymmetric mode is characterized by the out-of-phase charge distribution, indicated by the narrow band absorption peak of the absorption spectrum in Fig. 3a. Generally, the excitation of waveguide mode in plasmonic structures can restrict incident light to be stored in the waveguide layer for a long time], which would normally give rise to high energy dissipation and radiative damping corresponding to a distinct reduction of transmission and an increase of line width due to the intrinsic ohmic loss of metal. However, the transmission band of EIT resonance in the proposed plasmonic structure can achieve the efficiency of nearly 92% and the linewidth of only 0.14 nm, which is a seemingly counterintuitive finding. This is because the plasmonic field is canceled out due to the destructive interference of super-radiative nanowire dipolar and TM waveguide mode. This cancellation brings out the weak local field and low absorption around the metal nanowires. Figure 2b shows that the spatial distribution of total electric field in the structure for the transmission peak at λ = 1201.1 nm. The electric field is mainly localized around the ambient air areas and avoids most of the metal nanowires region. The field distribution for waveguide mode along the *z* and *x* directions is an obvious characteristic of guided TM mode. Furthermore, the quadrupolar charge distribution of each nanowire pair at the transmission peak is demonstrated in Fig. 2d, which further suppresses the radiative damping and absorption of the metal nanowires. This is also an abundant proof for the aforementioned destructive interference between the two excitation pathways.

In the previous studies^9, 18, 19, 27^, structural asymmetry is a crucial structural parameter to determine the optical properties of double-layer structures. Notably, in the following analysis, the coupling between nanowire pairs can be divided into two categories: weak coupling for the lateral shift *d*_*x*_ ≥ 450 nm (non-overlapping between two nanowires) and strong coupling for the lateral shift *d*_*x*_ < 450 nm. In Fig. 3, the influence of the lateral shift *d*_*x*_ on transmission, reflection and absorption spectra is plotted for the bilayer plasmonic waveguide structure. Only the lateral shift *d*_*x*_ is modified and other parameters are kept constant. The tunability of optical spectra response becomes evident with the decrease of the lateral shift *d*_*x*_. The most striking features are the increase of sideband transmission with the lateral shifts *d*_*x*_ < 450 nm, and the sudden drop of the efficiency of transmission peak with the lateral shifts *d*_*x*_ ≤ 150 nm. When the lateral shifts *d*_*x*_ is reduced to 450 nm, the coupling between nanowire pairs results in a strong redshift of the first-order symmetric mode generating EIT effect, the energy of which is lower than that of the antisymmetric one. Furthermore, the additional plasmon mode induces a transmission dip at the wavelength of 920 nm, leading to the formation of its right broad transmission peak and therefore results in the increased sideband transmission efficiency of shorter wavelength range of EIT phenomenon. The charge distribution of the additional plasmon mode, as presented in Fig. 3b, shows the characteristic of the second order antisymmetric mode, which is different from previous reported magnetic resonance based on strong near-field coupling^18, 19^ because there is almost no overlapping between the two nanowires in the *x* direction. When the lateral shift *d*_*x*_ is less than 450 nm, the two nanowires in a unit cell begin to overlap, and the near-field strong coupling between which gradually strengthens, resulting in the excitation of magnetic resonant mode in the overlap region of two interacting nanowires. With the decrease of lateral shifts *d*_*x*_, the antisymmetric mode from weak coupling is converted into the magnetic resonant mode from strong coupling. As demonstrated in Fig. 3c, the enhanced near-field interaction between the nanowires results in two distinct transmission dips at the wavelength of 840 nm and 1495 nm, which is different from the above mentioned first and second-order antisymmetric modes with *d*_*x*_ = 450 nm. The transmission dip at λ = 1495 nm is characterized by the out of phase oscillation of the free electrons in the gap between nanowires, which induces a circular current, directly resulting in a resonantly enhanced magnetic field. The magnetic field distribution of λ = 1495 nm in the inset of Fig. 3c shows a maximum of magnetic field in the center of overlapping gap between adjacent nanowires, which is identified as being the first order magnetic resonance^28, 29^. Therefore, the coupling of nanowire pairs is dominated by strong coupling magnetic resonance instead of weak coupling antisymmetric mode. While the charge and field distribution at the wavelength of 840 nm is completely different from the first order magnetic resonance, which demonstrates much richer space variation. The charge distribution at λ = 840 nm exhibits two current loops oscillating in opposite directions, leading to two maxima of magnetic field. Therefore, this resonance is regarded as the second order magnetic resonance, the characteristic of which can be clearly observed in the charge and field distribution of Fig. 3c. Compared to the case of the lateral shift *d*_*x*_ = 450 nm, the left sideband transmission efficiency of EIT effect at *d*_*x*_ = 300 nm is higher, just due to the excitation of the second order magnetic resonant mode. As the structural asymmetry is further decreased to *d*_*x*_ = 150 nm, a striking observation in Fig. 3d is the vanishing of narrow transmission band because of the altered near-field coupling between nanowires. As the overlapping region between nanowires increases, the magnetic resonant mode is gradually strengthened and has a redshift. The first order magnetic mode exceeds the scope of current wavelength range with *d*_*x*_ = 150 nm, while the resonant wavelength of the second order magnetic mode is shifted to 1328 nm. Similar to the case of *d*_*x*_ = 300 nm at λ = 840 nm in Fig. 3c, the characteristic feature of the second order magnetic mode can be clearly observed, and the field is strongly enhanced, as shown in the inset of Fig. 3d. At this moment, the super-radiative dipolar mode of nanowire pairs is absolutely replaced by second order magnetic mode, resulting in the disappearance of narrow transmission band and only a kink at λ = 1200 nm due to Rayleigh anomaly. The optical response of the aligned (*d*_*x*_ = 0 nm) double layer structure is also shown in Fig. 3e, exhibiting only a kink of Rayleigh anomaly. This is because the second order magnetic mode is optical inactive in perfectly aligned geometries without structural asymmetry. As a reference, the optical response spectra of single layer nanowire waveguide structure are shown in Fig. 3f. There is a narrow transmission peak accompanied by a spectrally broad nanowire dipolar mode indicated by the inset of Fig. 3f. Similarly, this narrow transmission peak arising from the destructive interference of dipolar mode of single nanowire and TM guided mode.

**Figure 4:**
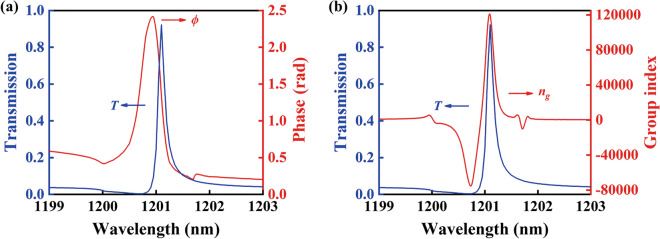
Slow light effect of bilayer plasmonic waveguide structure. (**a**) Calculated phase change (red line) is shown together with transmission spectrum (blue line). (**b**) The resulting group index around transmission peak.

Due to its sharp transmission peak and more than 92% transmission efficiency, the proposed plasmon-waveguide structure can be applied as high-performance slow light device, generally interpreted as incident light traversing the structure at a low group velocity. Figure 4 shows the slow light effect of the bilayer plasmonic waveguide structure. Figure 4a demonstrates the calculated transmission spectra and corresponding phase change with the lateral shift *d*_*x*_ = 550 nm fixed and other parameters keep constant, where a sharp transmission peak is accompanied by dramatic phase change of nearly 3π/4. On basis of the dispersion of phase in Fig. 4a, the dependence of group index *n*_*g*_ on the wavelength is calculated and depicted in Fig. 4b. It is clearly observed that the resulting group index *n*_*g*_ is highest at the location of transmission peak, which is determined by the tremendous change of the phase. A group index of 1.2 × 10^5^ in the proposed double-layer structure is nearly 50 times higher than that of the previously reported structure^19^, which implies that light traversing the entire structure takes a long time in comparison with light in the vacuum.

**Figure 5:**
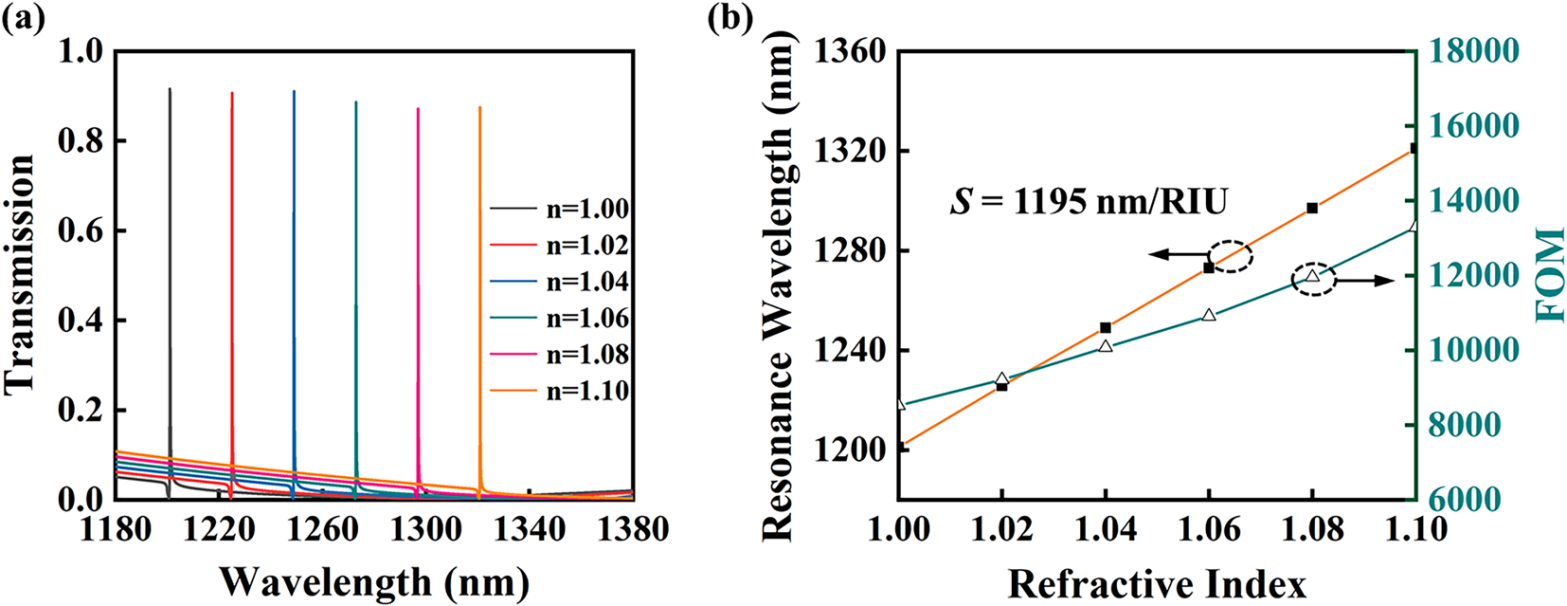
Refractive index sensing performance. (**a**) Transmission spectra of bilayer plasmonic waveguide structure for various ambient refractive indexes. (**b**) Dependence of extracted wavelength position of transmission peak and sensing figure of merit on environmental refractive index.

The proposed plasmonic-waveguide structure also has enormous potential for high performance sensing application due to the narrow line width for high signal-to-noise ratio and high refractive index sensitivity to surrounding medium. To quantitatively evaluate the sensing performance of the double layer plasmonic waveguide system, refractive index (RI) sensitivity and figure of merit (FOM) are frequently chosen as two crucial criteria. The refractive index sensing performance is shown in Fig. 5. Figure 5a shows the dependence of transmission spectra of the proposed structure on the refractive index of ambient environment. The extremely narrow band of the transmission spectra is not changed and keeps consistently high transmission efficiency and low sideband transmission with the increase of the ambient refractive index. Refractive index sensitivity (*S*) is defined as the ratio of the wavelength shift of transmission peak to the change of refractive index, which can be determined by the slope of linear fitting curve in Fig. 5b. So, the resulting refractive index sensitivity of 1195 nm/RIU is obtained for the proposed structure, which is higher than that of the previously reported plasmonic sensors^30, 31^. Another evaluation criterion, FOM, is identified as the ratio of RI sensitivity and the linewidth of transmission peak obtained by Lorentz fitting, and is a crucial metric to determine the performance of an optical sensor. The calculated FOM value is achieved up to 8500 at air and gradually increases with the increasing refractive index of surrounding environment, which is two orders of magnitude higher than that of the previously reported plasmonic sensors^32–34^. It is worth noting that, when the finite silica waveguide is replaced by silicon nitride waveguide with other parameters remaining constant, the double layer plasmonic waveguide system would also be a promising candidate for aqueous solution detection. The above sensing performances indicate that the plasmonic waveguide structure can be applied as a high-performance sensing detection platform.

## Conclusion

In conclusion, we have demonstrated an extremely narrow transmission band within a broad low-transmission region that supported in the asymmetric nanowire waveguide structure, arising from the destructive interference of super-radiative dipolar mode from weak coupling of nanowire pairs with an optical waveguide mode. The narrow transmission window is also benefited substantially from thin waveguide thickness in symmetric waveguide structure due to the absence of substrate, which has no cut-off thickness for the excitation of guided mode. Especially, it has been demonstrated that the lateral overlapping between two interacting nanowires allows for the excitation of strong coupling magnetic resonance mode, which seriously damages the low-efficiency sideband of the narrow transmission band, and even leads to the disappearance of the transmission window. Furthermore, the feasibility for slowing down light and high sensitivity sensing detection is also in principle verified for the plasmonic waveguide structure, which implies that it may be a potential candidate for developing high performance integrated nano-optical devices.
